# Surgical Management of a Large Radicular Cyst in a Maxillary Incisor Following Failed Regenerative Endodontics and Apical Plug in an Adolescent: A Case Report

**DOI:** 10.1155/crid/6691062

**Published:** 2026-03-11

**Authors:** Saeed Asgary

**Affiliations:** ^1^ Iranian Center for Endodontic Research, Research Institute of Dental Sciences, Shahid Beheshti University of Medical Sciences, Tehran, Iran, sbmu.ac.ir

**Keywords:** calcium derivative, dental radiography, endodontics, incisor, microsurgery, mineral trioxide aggregate, odontogenic cyst, periapical periodontitis, tooth resorption, volume CT

## Abstract

This case report describes the surgical treatment of a large radicular cyst in a maxillary incisor of an adolescent, following an unsuccessful regenerative endodontic procedure (REP) and an apical plug. It highlights minimally invasive, age‐appropriate decision‐making. A 14‐year‐old boy presented with an acute abscess (pain, swelling, and fever) in the right maxilla. Tooth #11 was nonvital, with apical resorption, an open apex, and a huge radiolucent lesion involving Tooth #12, confirmed by CBCT. Initial treatment included incision/drainage, calcium hydroxide dressing, and antibiotics, which resolved symptoms. REP was performed 3 months after presentation (inducing a blood clot, placing biomaterial, and coronal sealing). Partial healing was noted at 8 months, but lesion recurrence occurred asymptomatically at 19 months. An apical plug using calcium‐enriched mixture (CEM) was inserted, with slight extrusion observed. Despite patient comfort, follow‐ups at 37 and 44 months showed persistent nonhealing; CBCT revealed a 12 × 18 mm lesion, CEM extrusion in Tooth #11, and resorption defects. Because of the failure of nonsurgical endodontic treatments and the patient now being 18 years old, surgical intervention was performed. This involved mucoperiosteal flap elevation, enucleation of the cystic lesion, without resecting the root‐end, followed by root‐end preparation and filling/sealing with CEM cement in Tooth #11 and the vital #12 (due to cystic involvement). A bone substitute was also placed. Histopathology confirmed a radicular cyst. One year after surgery, radiographic healing and bone regeneration were complete, and the tooth remained functional and asymptomatic. This case highlights that extensive cystic pathology may limit the predictability of REP in certain extensive cystic lesions and supports staged surgical intervention after conservative methods fail at skeletal maturity.

## 1. Introduction

The management of nonvital immature permanent teeth with open apices presents a significant endodontic challenge. The primary goals are to eliminate infection, promote periapical healing, and ideally, facilitate continued root development to enhance long‐term tooth survival and function [1]. Traditional apexification techniques, involving long‐term calcium hydroxide dressings or the creation of an artificial apical barrier with materials such as mineral trioxide aggregate (MTA) or calcium‐enriched mixture (CEM) cement, effectively achieve a seal but do not promote further root maturation, leaving the tooth susceptible to fracture due to thin dentinal walls [2].

Regenerative endodontic procedures (REPs) have emerged as a biologically based alternative, which is aimed at promoting healing and ingrowth of vital connective tissue within the canal space, rather than true histological regeneration of the original pulp–dentine complex; the high tooth survival rate and patient satisfaction associated with these outcomes support REPs′ efficacy as a reliable treatment option [3, 4]. REP protocols typically involve minimal or no mechanical instrumentation, disinfection with low‐concentration irrigants (e.g., 1.5%–3% NaOCl, 17% EDTA), induction of apical bleeding to form an intracanal blood clot (acting as a scaffold rich in stem cells and growth factors), and placement of a biocompatible coronal seal over this matrix. Although REPs offer the potential for true regeneration and root maturation, outcomes are variable, and outcomes remain variable and may be influenced by the biological microenvironment, including inflammatory burden and epithelial activity, rather than lesion size alone [5].

Minimally invasive endodontic approaches prioritize the preservation of natural tooth structure, minimization of iatrogenic damage, and the use of biologically active strategies [6]. This philosophy is particularly crucial in adolescent patients, where preserving structural integrity and the potential for continued development are paramount. REPs inherently align with this principle by avoiding aggressive canal preparation. Similarly, apical plug techniques represent a minimally invasive strategy compared with traditional apexification, focusing solely on creating a seal at the apex without attempting to fill the entire canal [2].

Apical lesions associated with immature necrotic teeth encompass a biological spectrum ranging from granulomatous inflammation to true radicular cysts. Inflammatory radicular cysts represent a distinct pathological entity characterized by epithelial proliferation driven by chronic inflammatory signaling. Molecular evidence indicates that epithelial activation, rather than lesion size alone, plays a key role in cyst persistence and resistance to healing, which may influence outcomes of conservative endodontic approaches [5, 7].

When conservative endodontic therapies (REPs and apical plugs) fail to resolve infection or achieve periapical healing, surgical endodontics becomes necessary [8]. This involves accessing the root apex directly via a mucoperiosteal flap, thoroughly curetting the pathological periapical tissue, performing root‐end resection, preparing a retrograde cavity, and placing a root‐end filling biomaterial (e.g., MTA, CEM, and bioceramics) [9, 10]. Although traditionally considered a last resort, especially in young patients due to concerns about root length and surgical morbidity, advancements in techniques (microsurgery, magnification, biocompatible materials, etc.) and bone grafts have improved outcomes and reduced invasiveness.

This case report details the complex management of an immature maxillary central incisor in a 14‐year‐old boy with a large periapical lesion initially treated with a minimally invasive strategy (REP followed by an apical plug) that ultimately failed. It highlights the challenges associated with large lesions potentially cystic in nature, the limitations of REP in such scenarios despite symptomatic relief, the critical role of long‐term follow‐up, and the successful transition to definitive surgical management upon the patient reaching skeletal maturity, incorporating enucleation of a confirmed radicular cyst and root‐end surgery.

This case report was prepared in accordance with the CARE guidelines for case reports [11], as recommended by the EQUATOR Network.

## 2. Case Presentation

A 14‐year‐old boy, a healthy Iranian adolescent with no significant medical history or systemic conditions, presented with an acute abscess in the right anterior maxilla, characterized by severe pain, localized swelling, erythema, tenderness to palpation, and fever (> 38.5°C). He had recently completed orthodontic treatment (1 month before) and had a composite restoration on the upper right central incisor (#11) at the same time.

Clinical examination revealed no caries or fractures, but Tooth #11 was nonresponsive to cold and electric pulp sensibility tests, whereas adjacent teeth (#12, lateral incisor; #13, canine; and #21, left central incisor) responded normally. A periapical radiograph and an orthopantomogram (OPG) (Figure 1a,b) demonstrated apical root resorption in #11 and #12, open apices, and an extensive radiolucent lesion (~20 × 20 mm). Cone‐beam computed tomography (CBCT) (Figures 1c, 1d, 1e, 1f, 1g, 1h, 1i, 1j, and 1k) confirmed a well‐defined hypodense lesion enveloping the apices of #11 and #12, extending to the mesial aspect of #13′s apical root third without direct apex involvement. The final diagnosis was an acute apical abscess secondary to pulp necrosis/infection in #11.

Figure 1Initial diagnostic imaging of the maxillary anterior lesion. (a) Periapical radiograph revealing apical root resorption and open apices in Teeth #11 and #12, associated with a large radiolucent lesion. (b) OPG demonstrating lesion extension to just beneath the nasal floor, with the apex of Tooth #12 located centrally within the radiolucent area. (c) CBCT reconstructions corroborating periapical and OPG findings. (d–f) Sagittal views of Tooth #11 confirming an extensive lesion encasing the apex, accompanied by external root resorption, open apex, and buccal cortical plate perforation; Tooth #12 exhibits external root resorption predominantly on the distal aspect of the apex; apex of Tooth #13 remains uninvolved. (g) Coronal view delineating the lesion margins. (h–k) Axial slices illustrating both mesial extension and buccal expansion of the lesion.

**Figure:**
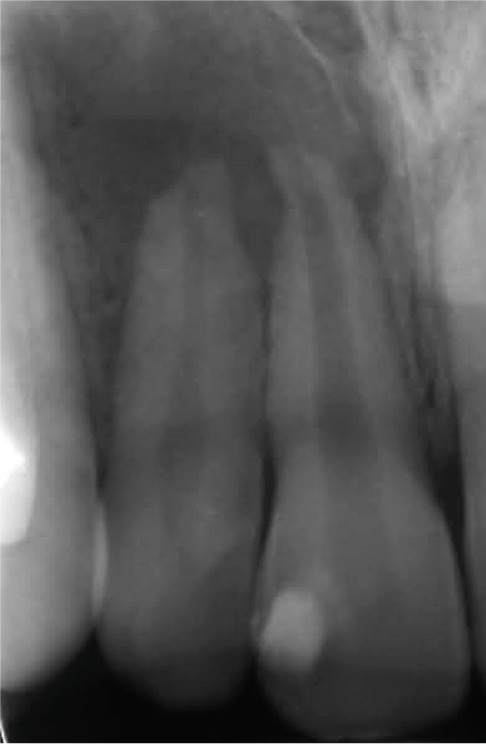
(a)

**Figure:**
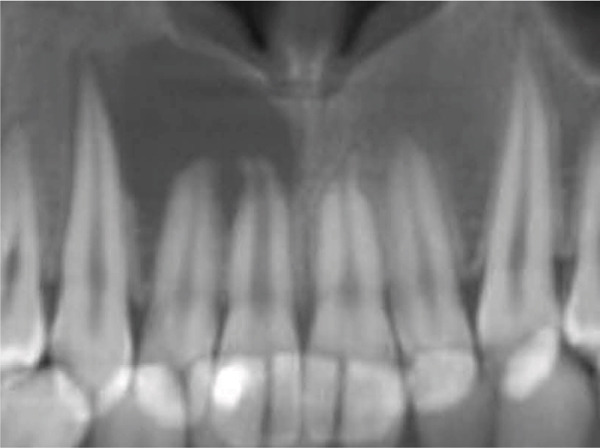
(b)

**Figure:**
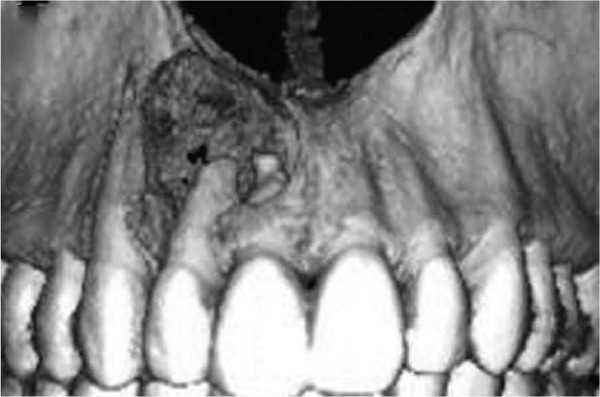
(c)

**Figure:**
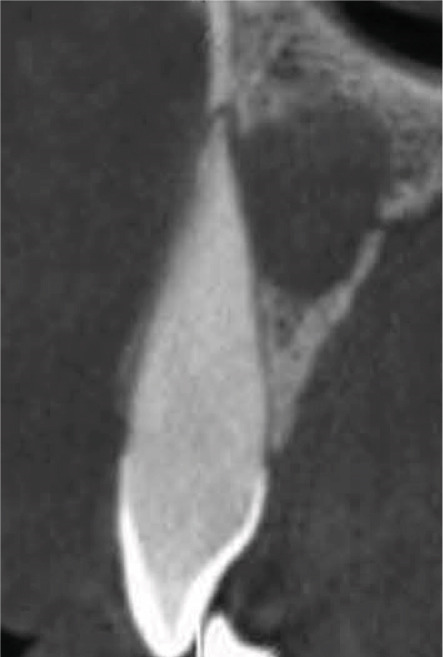
(d)

**Figure:**
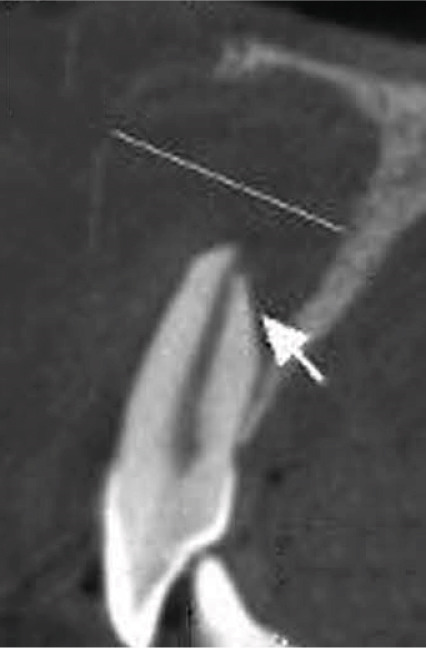
(e)

**Figure:**
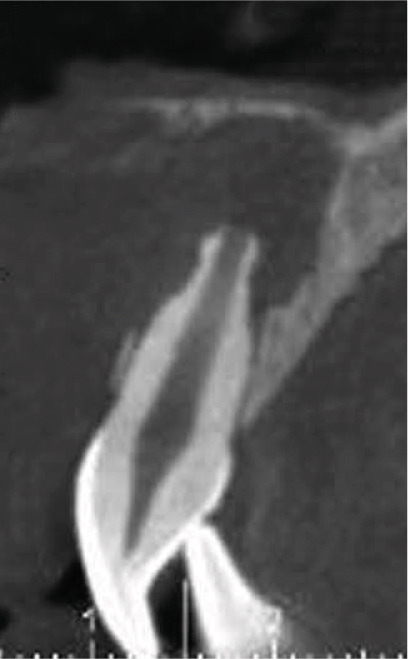
(f)

**Figure:**
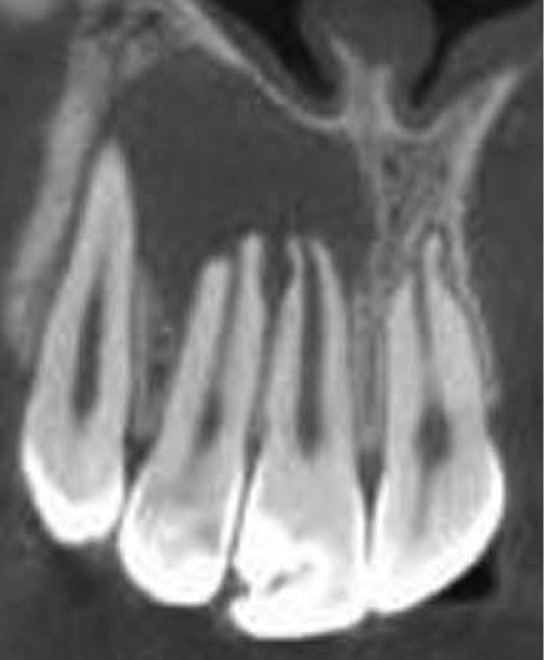
(g)

**Figure:**
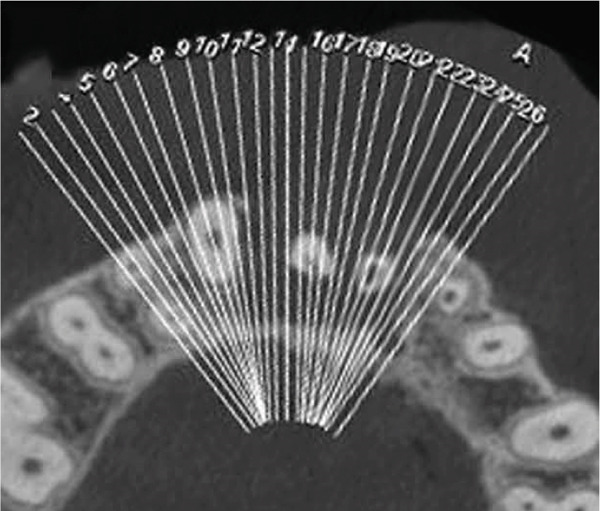
(h)

**Figure:**
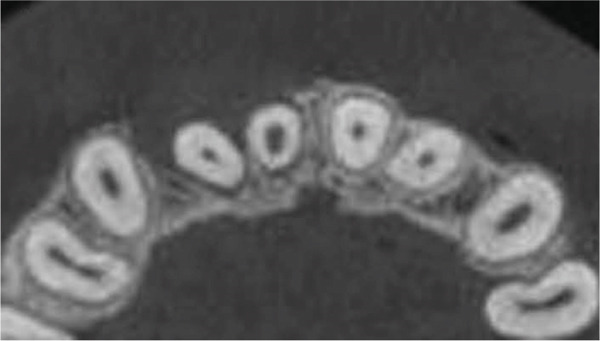
(i)

**Figure:**
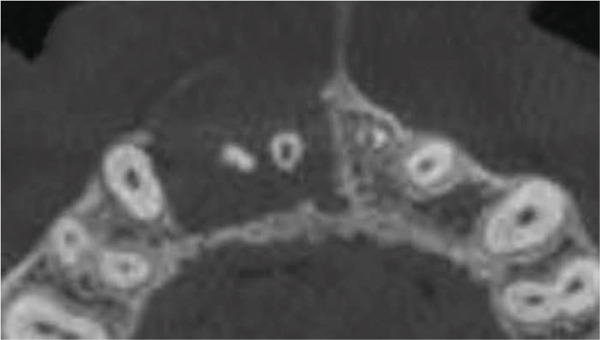
(j)

**Figure:**
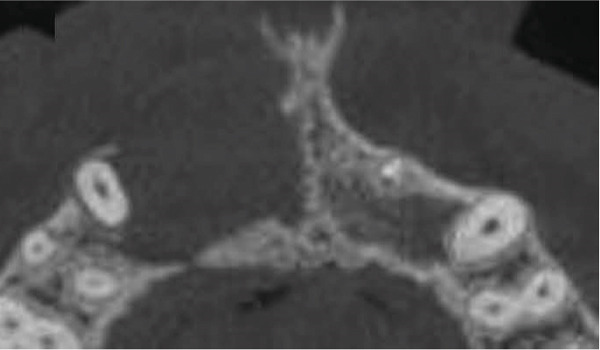
(k)

Considering the patient′s age and a minimally invasive approach, the REP was initiated immediately after informed parental consent. Despite the lesion′s large size and well‐defined borders—which may suggest, but do not confirm cystic pathology [7]—the REP was chosen in light of the patient′s young age, skeletal immaturity, and parental preference for a minimally invasive approach, consistent with the principle of attempting conservative options before surgical intervention. Current molecular evidence indicates that even large periapical lesions with radiographic features of inflammatory radicular cysts may respond to nonsurgical treatment, supporting a conservative first‐line approach when clinically feasible [5]. Moreover, several previous reports have demonstrated successful resolution of even very large periapical lesions with REPs, supporting this treatment choice in selected cases [12, 13].

Incision and drainage were performed via the access cavity, yielding heavy purulent exudate. The canal was gently instrumented, dressed with calcium hydroxide (Figure 2a), and amoxicillin (500‐mg TID for 7 days) was prescribed. Symptoms resolved within 24 h, with complete resolution at 1 week. The patient returned 3 months later (Figure 2b) for completion of REP: The canal was irrigated with 20 mL of 1.5% NaOCl followed by 10 mL of 17% EDTA, each with a 1‐min contact time and final saline flush. Then a blood clot was induced, and the clot was capped with CEM cement, followed by a glass ionomer cement (GIC) coronal seal (Figure 2c).

Figure 2Regenerative endodontic procedure (REP). (a) Postinitial visit: calcium hydroxide dressing in Tooth #11 (March 4, 2019). (b) Pre‐REP: persistent radiolucency before blood clot induction. (c) Immediate post‐REP: CEM cement barrier and GIC coronal seal. (d–e): 8‐ and 14‐month follow‐ups: partial healing with near‐normal PDL around Teeth #11–#12.

**Figure:**
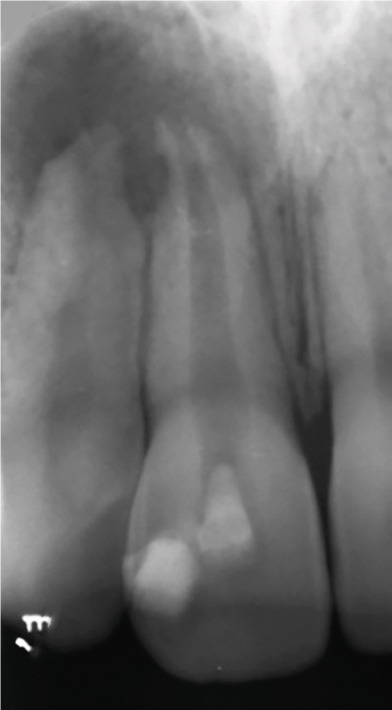
(a)

**Figure:**
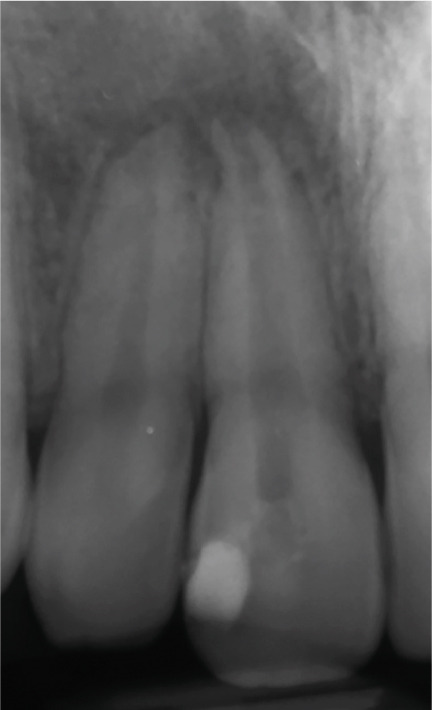
(b)

**Figure:**
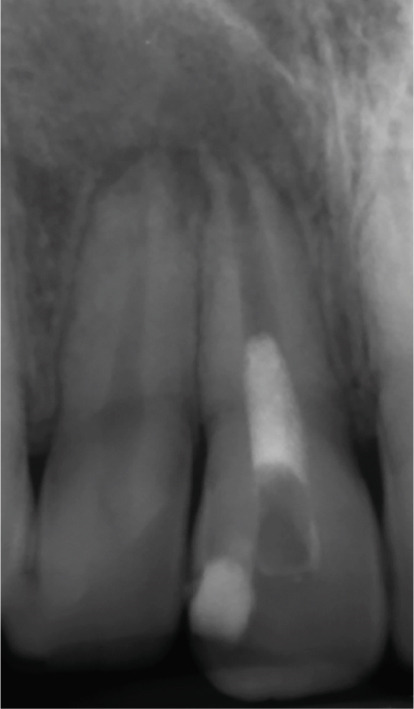
(c)

**Figure:**
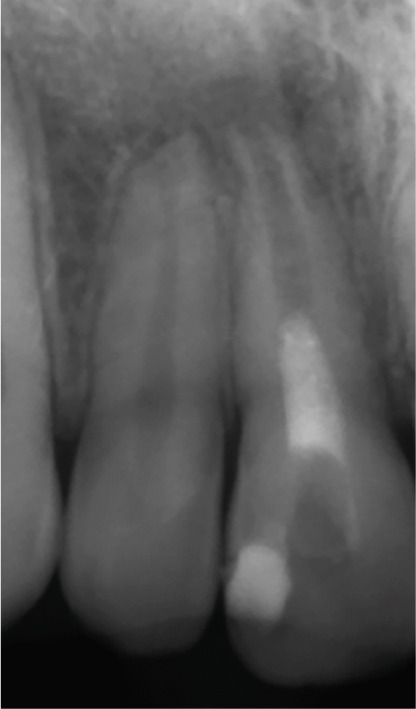
(d)

**Figure:**
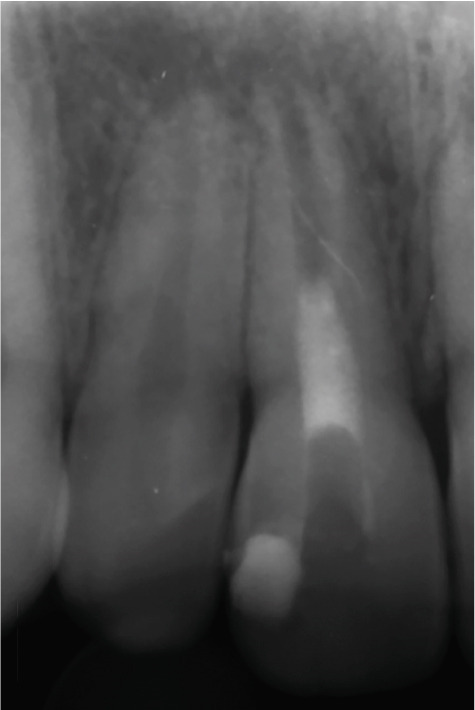
(e)

At the 8‐ and 14‐month follow‐up (Figure 2d,e), partial healing of the lesion was evident radiographically, with no symptoms and a nearly normal periodontal ligament (PDL) space around Tooth #12. However, root lengthening/thickening of Tooth #11 was absent. The patient remained asymptomatic until 16 and 19 months after the initial presentation (Figure 3a,b), when radiographs revealed lesion recurrence without clinical symptoms. REP was deemed unsuccessful, and an apical plug with CEM cement was placed after removal of the canal content (Figure 3c,d). The material extruded slightly beyond the apex (~1 mm), suggesting a possible root canal perforation due to inflammatory resorption. Follow‐ups at 37 months (Figure 3e) and 44 months (Figure 3f) showed no radiographic healing, though the patient remained asymptomatic.

Figure 3Apical plug and long‐term follow‐up. (a) 16‐month follow‐up: asymptomatic recurrence. (b) 19‐month recurrence: enlarged radiolucency. (c) Before apical plug insertion: complete removal of CEM cement from the root canal of Tooth #11. (d) Immediately after apical plug insertion: CEM placement with slight apical extrusion. (e) 37‐month follow‐up: nonhealing. (f) 44‐month follow‐up: persistent lesion.

**Figure:**
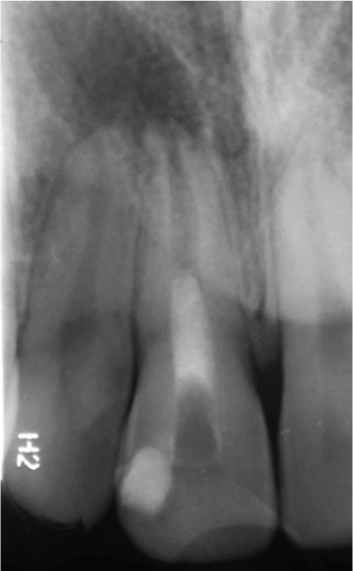
(a)

**Figure:**
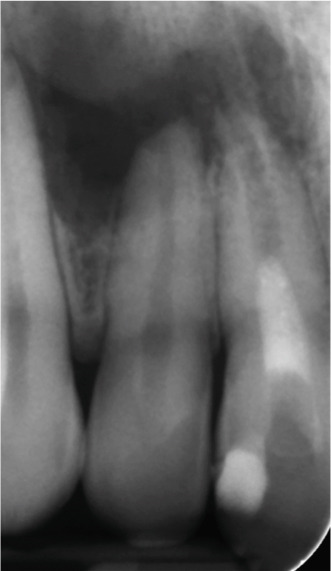
(b)

**Figure:**
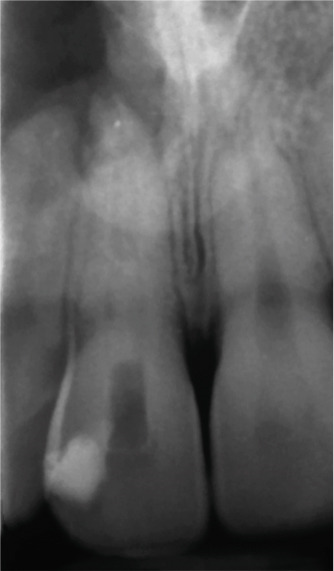
(c)

**Figure:**
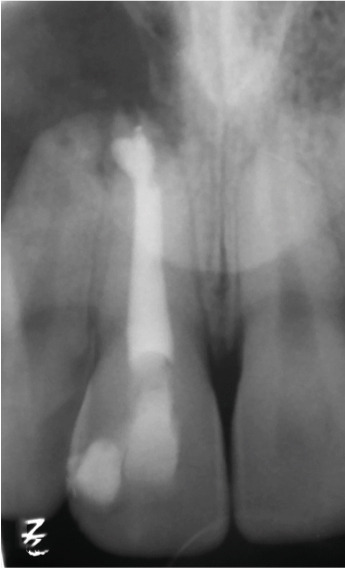
(d)

**Figure:**
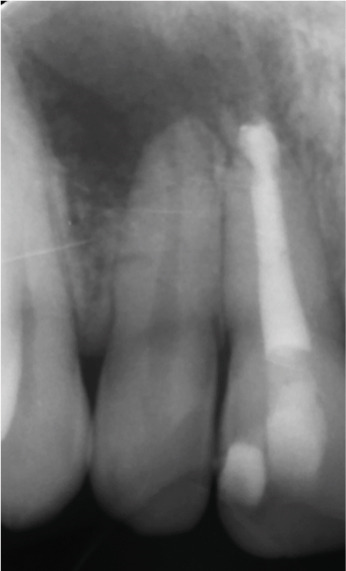
(e)

**Figure:**
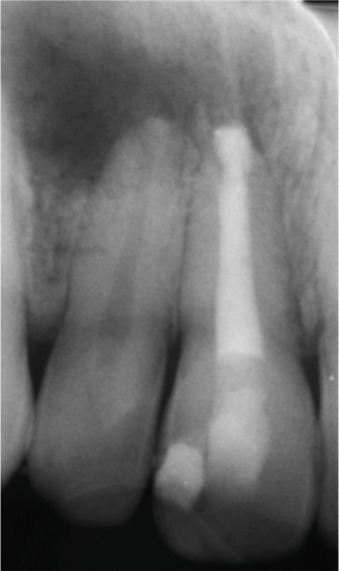
(f)

Surgical intervention was deferred, whereas the patient remained asymptomatic, allowing continued monitoring and completion of conservative treatment attempts before definitive surgical management. Periapical radiographs were used for interim monitoring to limit radiation exposure, whereas CBCT was reserved for initial diagnosis and presurgical evaluation.

Subsequent CBCT (Figures 4a, 4b, 4c, 4d, 4e, 4f, 4h, 4i, and 4j) identified a persistent 12 × 18 mm lesion around Teeth #11 and #12, with a densely filled apical canal segment in #11 and CEM extrusion (2 × 2 mm) due to inflammatory resorption. The lateral incisor (#12) and canine (#13) were tested, and they were vital.

Figure 4CBCT of persistent pathology (postapical plug failure). (a–c) Sagittal and axial slices of #11 showing dense apical filling, CEM extrusion (arrow), and inflammatory resorptive defect (star). (d–g) Coronal and sagittal views of the 12.3 × 18.5 mm lesion involving #11–#12 apices. (h) Dimensions of the lesion in an axial view. (i) Axial slice at 3 mm from apex: canal calcification and buccal CEM extrusion (arrow). (j) Axial slice near apex: unfilled main canal of #12 (arrow) due to resorption.

**Figure:**
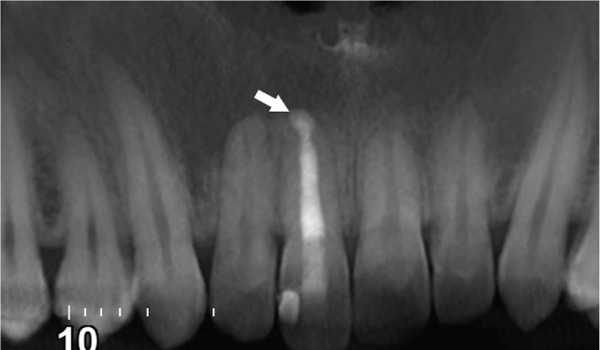
(a)

**Figure:**
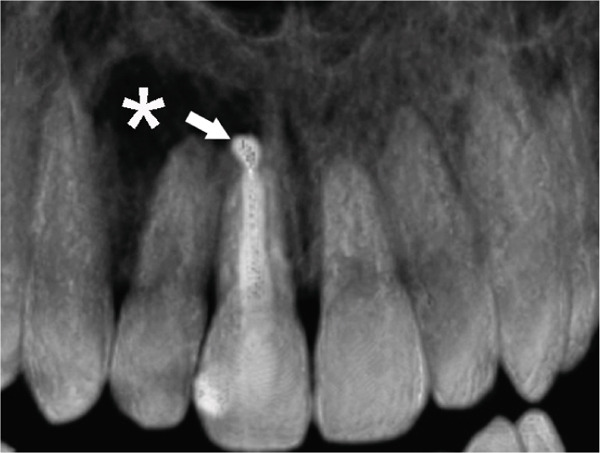
(b)

**Figure:**
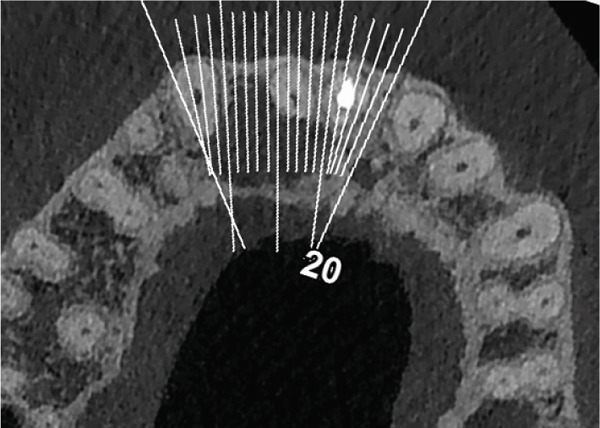
(c)

**Figure:**
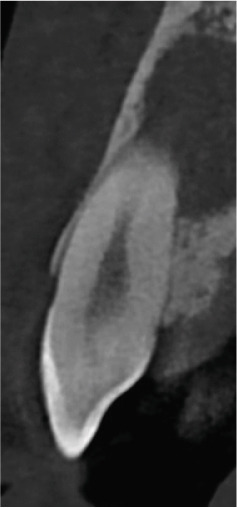
(d)

**Figure:**
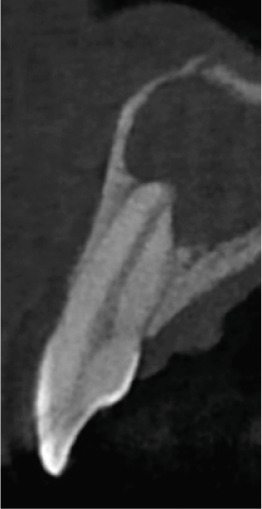
(e)

**Figure:**
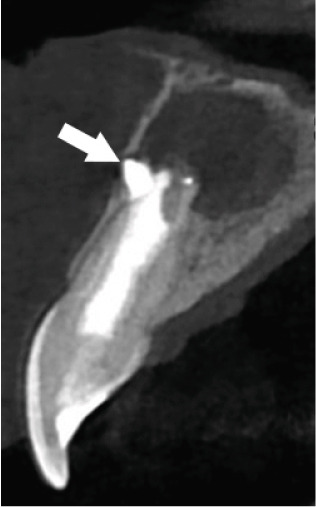
(f)

**Figure:**
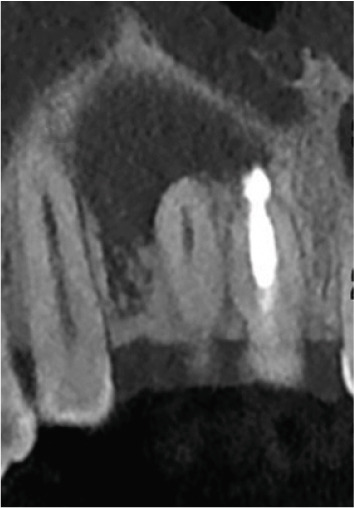
(g)

**Figure:**
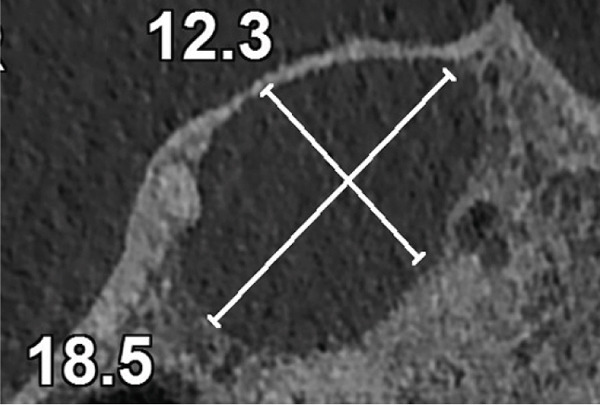
(h)

**Figure:**
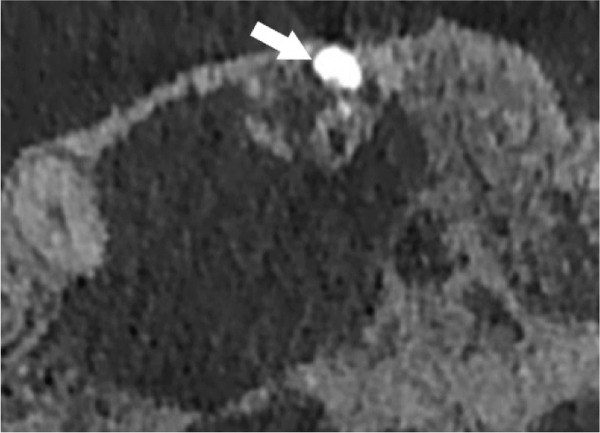
(i)

**Figure:**
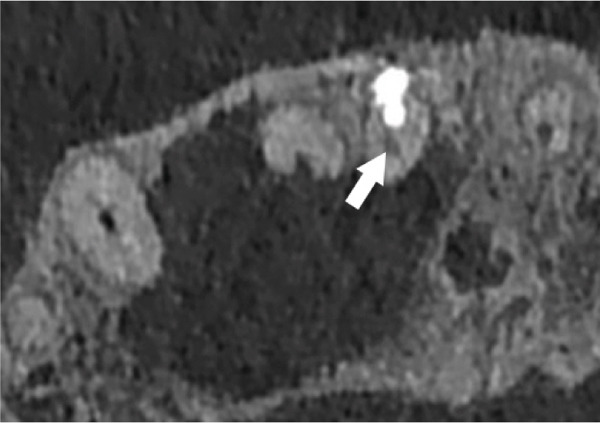
(j)

Given the failure of nonsurgical interventions and the patient now being 18 years old, surgical management was planned. Under local anesthesia, a full mucoperiosteal flap was elevated, revealing buccal cortical plate perforation and a large cystic cavity. En bloc excision of the cyst was performed, followed by thorough curettage. Root‐end resections were omitted to preserve root length, given preexisting resorption; retrograde preparation and CEM sealing were performed to address inaccessible canal complexities. The defect was grafted with bone substitute material (Cerabone, Botiss, Berlin, Germany), a xenogeneic deproteinized bovine bone mineral (DBBM); consent was obtained, and the flap was sutured.

Histologic analysis confirmed a radicular cyst, showing a cystic lumen lined by nonkeratinized squamous epithelium (Figure 5a). The fibrotic capsule wall exhibited chronic inflammatory infiltrate composed of lymphocytes and plasma cells, along with a dominant population of foam cells, indicating a chronic foreign body reaction (Figure 5b). Additionally, particles of extruded calcium–silicate cement were observed embedded within the inflamed connective tissue (Figure 5c).

Figure 5Histopathology of enucleated cyst. (a) Low magnification (H&E, ×40): cystic lumen lined by squamous nonkeratinized epithelium. (b) Medium magnification (H&E, ×100): fibrotic capsule with chronic inflammatory infiltrate consisting of lymphocytes and plasma cells, with a dominant population of foam cells. (c) High magnification (H&E, ×200): particles of extruded calcium–silicate cement embedded within inflamed connective tissue.

**Figure:**
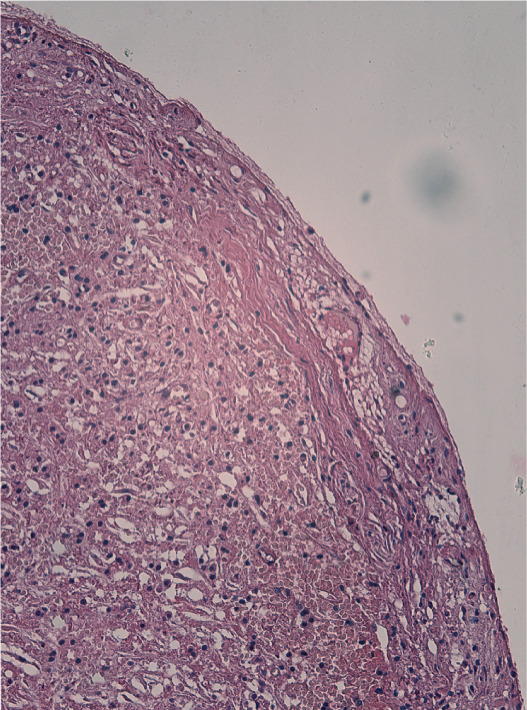
(a)

**Figure:**
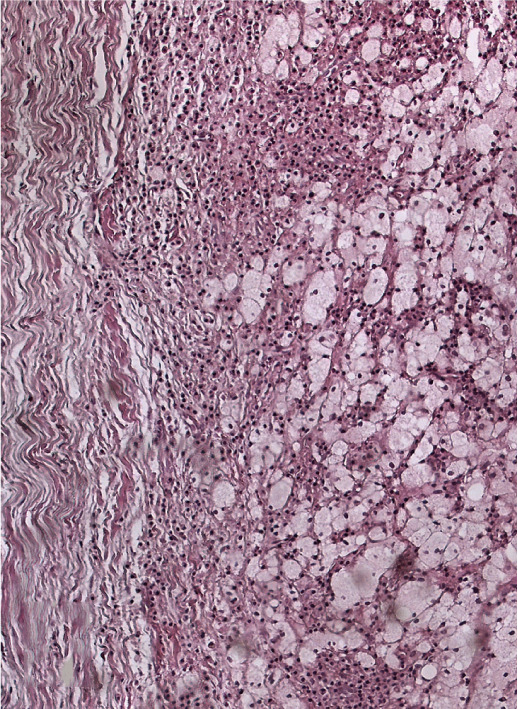
(b)

**Figure:**
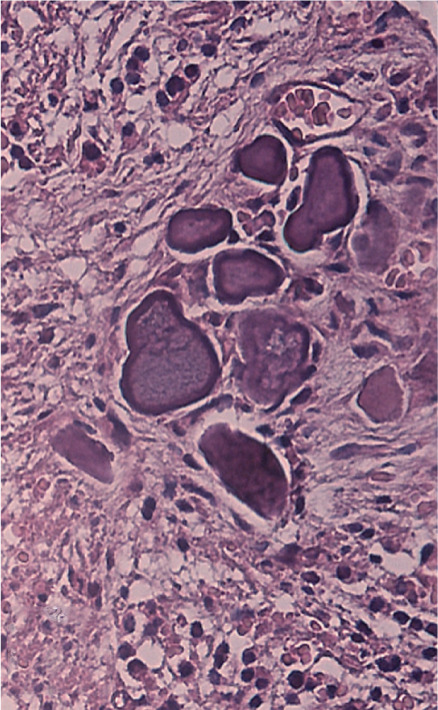
(c)

**Figure 7:**
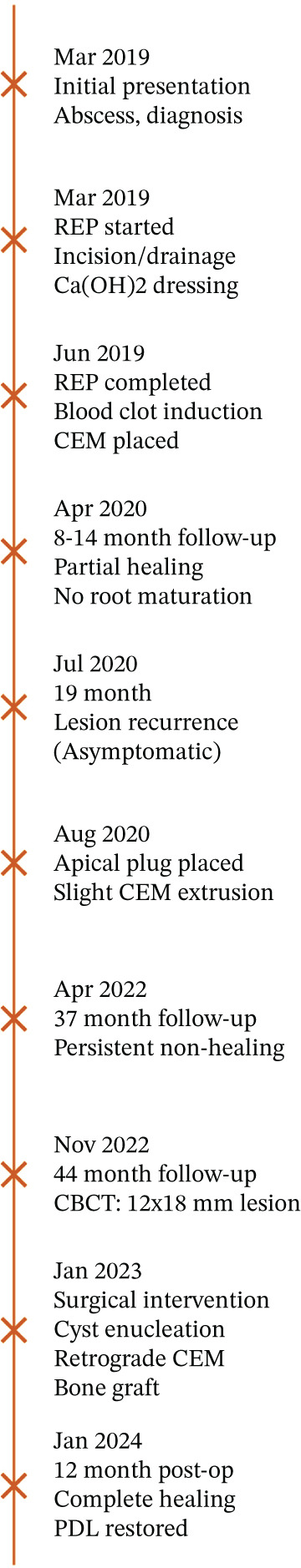
Timeline of clinical management and outcomes.

Immediate postoperative radiography (Figure 6a) confirmed adequate root‐end fillings. At the 1‐year follow‐up (Figure 6b), a favorable radiographic appearance consistent with healing and bone repair was observed, with the tooth asymptomatic and fully functional. A graphical timeline of the clinical sequence is presented in Figure 7.

Figure 6Surgical outcome. (a) Immediate postoperative radiograph: retrograde CEM fillings in #11 and #12 (arrows) with bone graft. (b) 12‐month follow‐up: complete bone regeneration, reestablished PDL space, and resolution of radiolucency.

**Figure:**
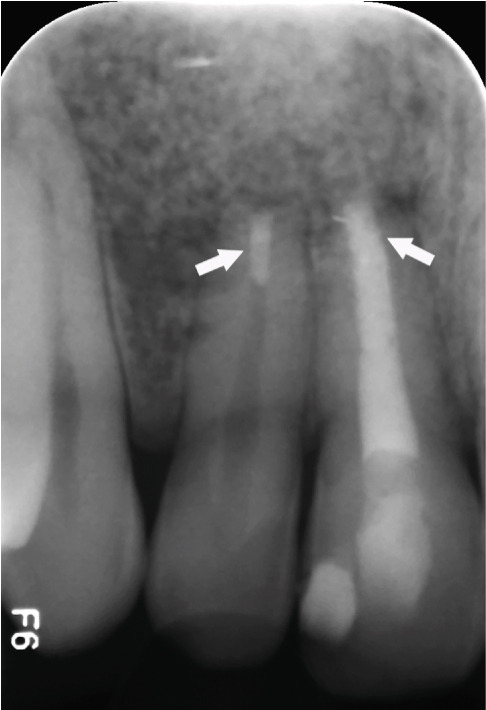
(a)

**Figure:**
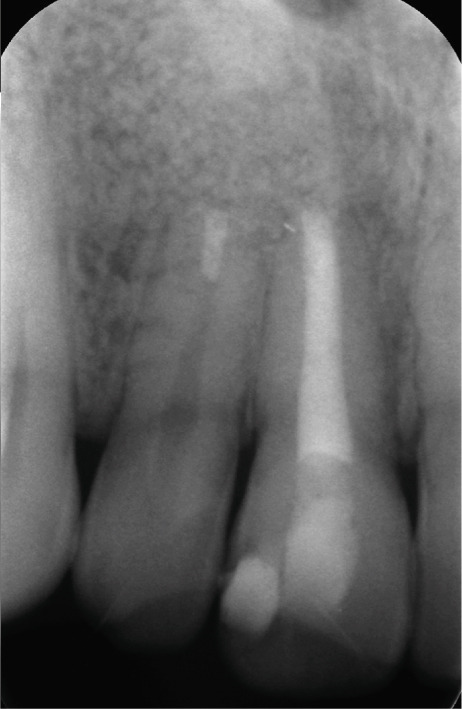
(b)

## 3. Discussion

This case illustrates the challenges of managing a large radicular cyst in an immature maxillary incisor after two conservative approaches: a REP and an apical plug with CEM cement, which were unsuccessful. Its uniqueness lies in the staged, long‐term management over nearly 5 years, progressing from REP to an apical plug and finally to surgical intervention, with all stages documented by detailed radiographic and histopathologic correlation. The histopathological confirmation of an inflammatory radicular cyst, with active epithelial lining and chronic inflammatory infiltrate, provides a biological explanation for the lesion′s persistence and resistance to conservative endodontic approaches. This course underscores the limitations of biologically based, minimally invasive protocols for extensive cystic pathology and emphasizes the complex decision‐making involved in treating adolescent patients with large endodontic lesions, ultimately necessitating a definitive surgical approach upon skeletal maturity. Although large cystic lesions are a relative contraindication for REPs, emerging evidence suggests that regenerative techniques can still induce healing [12, 13]. Therefore, the initial attempt at REP was justified by the patient′s youth, incomplete skeletal development, the potential for further root maturation, and the principle of exhausting less invasive approaches before surgical intervention.

REP protocols are aimed at promoting repair through ingrowth of vital connective tissue and revascularization of the canal space, rather than true regeneration of the original pulp–dentine complex [14]. In this case, the initial REP led to temporary symptom resolution and partial radiographic healing; however, root maturation did not occur, and the lesion recurred asymptomatically at 19 months. The failure of REP in this instance was likely influenced by the lesion′s considerable size and cystic nature, which may have compromised the local microenvironment, including impaired vascular infiltration, persistent microbial contamination, and structural barriers imposed by the cystic epithelium. Additionally, potential biological limitations could include hindered angiogenesis, the persistence of an epithelial‐lined cystic cavity that restricts the movement of cells, and a prolonged inflammatory microenvironment that does not support predictable regenerative outcomes [15].

The subsequent orthograde apical plug placement with CEM cement also failed to achieve long‐term periapical healing, despite the material′s known sealing ability and biocompatibility [10]. Chronic inflammation associated with the cyst likely induced progressive apical root resorption, compromising the integrity of the apical barrier and facilitating extrusion of the apical plug material. Histological examination revealed the presence of extruded CEM cement particles surrounded by inflammatory cells. This finding should not be interpreted as evidence of a classical *foreign body reaction*, since calcium silicate–based biomaterials such as MTA and CEM are well documented to be biocompatible, bioactive, and capable of modulating the immune response to promote repair and hard tissue formation [10, 16]. The observed histological picture is consistent with a localized tissue response to extruded biomaterial rather than cytotoxicity/incompatibility. Indeed, CEM and other calcium silicate–based biomaterials are known to release calcium ions, induce alkalinity, and support angiogenesis and mineralization, all of which facilitate healing and regeneration. Therefore, the persistence of the lesion in this case is more reasonably attributed to the cystic pathology and complex apical resorption/anatomy rather than any inherent adverse reaction to the cement. CBCT imaging further confirmed the structural limitations that impeded success, revealing inaccessible canal anatomy due to inflammatory resorption and importantly, the unfilled apex of Tooth #11. The latter likely acted as a continuous portal of communication between the cystic cavity and the periapical tissues, perpetuating microbial leakage and inflammatory stimulus, and thereby undermining complete resolution [17].

Surgical endodontic intervention proved successful in this case, owing to several key factors. First, the enucleation of the cyst eliminated the principal source of persistent pathology by removing the epithelial lining and associated inflammatory mediators. A critical strategic decision was to avoid root‐end resection to preserve the remaining root structure already compromised by resorption. Instead, a reliable apical seal was achieved for both the nonvital central incisor and the vital lateral incisor through root‐end preparation and retrograde placement of CEM cement [10]. The decision to include the lateral incisor was based on its apex being encompassed by the cystic cavity. This provided a biologically sound alternative to orthograde root canal treatment, operating on the principle that a durable apical seal is sufficient to prevent microbial ingress and support bone healing once the communication to the periapical tissue is sealed with suitable endodontic biomaterial [18]. This approach achieves the primary objective of RCT: preventing reinfection while avoiding the structural compromise of full instrumentation, thereby reducing procedural complexity, chair time, and patient discomfort. The timing of surgery was determined by lesion persistence despite conservative management, patient compliance, and readiness for definitive intervention. The prolonged presurgical follow‐up was an intentional part of this strategy, balancing the management of persistent pathology with the benefits of delayed intervention.

The 1‐year follow‐up confirmed complete radiographic healing, reestablishment of a normal PDL space, and restoration of function, attesting to the success of the surgical approach. Recent long‐term evidence indicates that prognostic factors such as periodontal pocket depth ≥ 4 mm, apicomarginal bone defects, and molar tooth position are associated with reduced success rates of apical microsurgery for radicular cysts [19]. The favorable outcome can be attributed to the biocompatibility, bioactivity, and sealing efficacy of CEM cement, which has demonstrated reliable performance in retrograde applications, particularly in cases involving complex apical anatomy and compromised root structures [10]. This case reinforces the clinical value of a staged endodontic strategy, beginning with minimally invasive methods in younger patients and transitioning to surgical management when conservative treatments fail and anatomical and developmental conditions are optimal.

The patient reported satisfaction with the aesthetic and functional outcome and relief after definitive surgical management.

## 4. Conclusions

This case highlights the clinical limitations of REP and orthograde apical barrier techniques in managing large cystic lesions in immature permanent teeth. Histologically confirmed radicular cysts may create a biological environment incompatible with predictable regenerative outcomes due to epithelial persistence, chronic inflammation, and impaired angiogenesis. Although minimally invasive approaches remain appropriate as first‐line strategies in young patients, definitive surgical management may be required when conservative therapies fail. A staged, patient‐specific treatment strategy remains essential for optimizing outcomes in complex endodontic cases.

## Author Contributions

Saeed Asgary: conceptualization, methodology, clinical treatment, investigation, writing—original draft, writing—review and editing, and supervision.

## Funding

No funding was received for this research.

## Ethics Statement

Ethical approval was not required for this study, as it is a single retrospective case report. The report was prepared in accordance with institutional and journal ethical standards.

## Consent

Written informed consent for treatment and for publication of this case report was obtained from the patient after he reached 18 years of age.

## Conflicts of Interest

The author declares no conflicts of interest.

## Data Availability

The data that support the findings of this study are available from the corresponding author upon reasonable request.
